# Saponin Facilitates Anti-Robo1 Immunotoxin Cytotoxic Effects on Maxillary Sinus Squamous Cell Carcinoma

**DOI:** 10.1155/2020/9593516

**Published:** 2020-03-11

**Authors:** Noriko Komatsu, Miku Komatsu, Riuko Ohashi, Akira Horii, Kazuto Hoshi, Tsuyoshi Takato, Takahiro Abe, Takao Hamakubo

**Affiliations:** ^1^Department of Oral and Maxillofacial Surgery, The University of Tokyo Hospital, Hongo, Bunkyo-ku, Tokyo 113-8655, Japan; ^2^Department of Protein-protein Interaction Research, Institute for Advanced Medical Sciences, Nippon Medical School, Kosugi-cho, Nakahara-ku, Kawasaki 211-8533, Japan; ^3^Department of Molecular Patology, Tohoku University of School of Medicine, Seiryo-machi, Aoba-ku, Sendai, Miyagi 980-8575, Japan; ^4^Histopathology Core Facility, Niigata University Faculty of Medicine, Asahimachi-dori, Chuo-ku, Niigata 951-8510, Japan; ^5^Division of Molecular and Diagnostic Pathology, Niigata University Graduate School of Medical and Dental Sciences, Asahimachi-dori, Chuo-ku, Niigata 951-8510, Japan; ^6^JR Tokyo General Hospital, Yoyogi, Shibuya-ku, Tokyo 151-8528, Japan

## Abstract

Head and neck squamous cell carcinoma (HNSCC) is one of the most common cancers worldwide. The standard treatment of surgery, chemotherapy, and radiotherapy can result in long-term complications which lower the patient's quality of life, such as eating disorders, speech problems, and disfiguring or otherwise untoward cosmetic issues. Antibody therapy against cancer-specific antigens is advantageous in terms of its lesser side effects achieved by its greater specificity, though the antitumor activity is still usually not enough to obtain a complete cure. Robo1, an axon guidance receptor, has received considerable attention as a possible drug target in various cancers. We have shown previously the enhanced cytotoxic effects of saporin-conjugated anti-Robo1 immunotoxin (IT-Robo1) on the HNSCC cell line HSQ-89 in combination with a photochemical internalization technique. Considering the light source, which has only limited tissue penetrance, we examined the drug internalization effect of saponin. Treatment with saponin facilitated significant cytotoxic effects of IT-Robo1 on HSQ-89 cells. Saponin exerts its own nonspecific cytotoxicity, which may cover the actual extent of the internalization effect. We thus examined whether a flashed treatment with saponin exerted a significant specific cytotoxic effect on cancer cells. The combination of an immunotoxin with saponin also exhibited a significant tumor-suppressive effect on mice HSQ-19 xenografts. These results suggest the utility of saponin treatment as an enhancer of immunotoxin treatment in cancer.

## 1. Introduction

Head and neck squamous cell carcinoma (HNSCC) is the sixth most common cancer worldwide [1, 2]. The morbidity and mortality rates for HNSCC have changed little over the last 30 years [3]. In addition to the death rate, a major problem is that conventional treatments such as surgery, radiotherapy, and chemotherapy result in long-term functional decline, including eating disorders as well as speech and cosmetic problems resulting in a diminished quality of life (QOL) [4]. Thus, the development of novel treatments to minimize these treatment-related complications is an urgent issue. Monoclonal antibody treatment is one of the approaches expected to afford improved care. Cetuximab and Nivolumab have been approved for HNSCC treatment by the Food and Drug Administration (FDA) [5, 6]. However, the antitumor effects of antibodies under the aegis of antibody dependent cellular cytotoxicity (ADCC) have proven to be inadequate for solid tumors. To overcome this problem, many techniques to enhance the cytotoxic activity, such as antibody drug conjugates (ADCs), immunotoxins (ITs), and radioimmunotherapy (RIT) have been developed [7–9].

ITs or chimeric toxins are designed such they have a cancer surface antigen-specific portion and protein toxin portion. The transmembrane receptors are the molecular target of these drugs so that monoclonal antibodies against the receptor or the ligand peptide of the receptor are mostly used. Frequently the protein toxins that are used are bacterial toxins or ribosome-inactivating proteins of plant origin [8]. Saporin, which is isolated from the seeds of the plant *Saponaria officinalis*, is categorized as type I ribosome-inactivating protein (RIP) [10]. Saporin is approximately 30kd molecular weight. It does not have a natural cell binding domain, so it is active only when it is endocytosed with a receptor-specific molecule or similar mechanism and transferred into the cytosol [11]. The type I RIP is a very powerful toxin in cancer therapy, and the major problem with its use is the need to combine it with some means to transfer it from the endosome to the cytosol.

We previously tried a photochemical internalization (PCI) method [12, 13] of a saporin-conjugated anti-Robo1 antibody for delivery into cancer cells and found a several hundred times augmentations of cytotoxic activity of anti-Robo1 IT [14]. Thus, the PCI method has proven effective for achieving endosomal release of the IT. However, it has a limitation in that the light does not penetrate into the deep tissues.

On the other hand, the saponins also originate mainly from plants and are known to be surface-active glycosides with many biological effects on membrane permeabilization, cholesterol metabolism, immune system modulation, and cancer growth inhibition [15]. Among various common commercially available saponins, *Quillaja saponaria* saponin (*Quillaya* saponins) used in this study is the extract from the bark of the South American soaptree and is a heterogeneous mixture of molecules varying both in their aglycone and sugar moieties. *Quillaya* saponins exhibit various biological activities such as hemolytic, anti-inflammatory, immune-stimulatory, antiviral, and cytotoxic activities [16, 17]. The mechanism of IT internalization by means of saponin has been reported as due to the transposition of the toxin from the endosomes to the cytosol without affecting the plasma-membrane integrity [18–20].

Robo1 was initially discovered as an axon guidance receptor in *Drosophila* [21]. The Robo family consists of Robo1-4 [22]. Human Robo1 has five immunoglobulin-like domains and three fibronectin III-like domains in its extracellular portion [22]. Robo1 is known to be expressed in fetal tissues, especially in the nervous system, and was originally reported as a tumor-specific antigen in liver cancer [23]. It is now found in a wide range of cancers, such as colon, breast, pancreatic, and lung cancer, and squamous cell carcinoma of the head and neck [24–26]. Robo1 is also expressed in the endothelial cells of neoangiogenetic vessels in both neoplastic and nonneoplastic diseases [26]. It has been reported that the Slit2/Robo1 signal in cancer plays an important role in invasion, migration, the epithelial-mesenchymal transition, as well as tumor-induced angiogenesis [24, 25, 27]. We have developed an anti-Robo1 monoclonal antibody [28] and showed the antitumor effects of an isotope-labelled version of this antibody against hepatocellular carcinoma and small cell lung cancer xenografts [29, 30].

In this study, we reconfirmed that endosomal release is necessary for saporin-conjugated anti-Robo1 antibody (IT-Robo1) in the cytotoxic effect against the Robo1-expressing maxillary sinus SCC cancer cells HSQ-89 using saponin. We also checked whether saponin treatment exerts a synergistic effect on the antitumor activity of IT in HSQ-89 xenograft mice. The results suggest that saponin facilitates the endosomal release of IT. The drug delivery system described here should be applicable to other targets, thus widening the therapeutic window for refractory cancers.

## 2. Materials and Methods

### 2.1. Cells

The HNSCC cell line HSQ-89 (derived from the maxillary sinus RCB0789) was purchased from RIKEN (Saitama, Japan). In a previous study, we confirmed the expression of Robo1 mRNA and protein in HSQ-89 cells by reverse transcription real-time PCR, Western blot analysis, and flow cytometry [14]. HSQ-89 cells were cultured in Dulbecco's Modified Eagle's Medium (DMEM) (Sigma Aldrich, MO, USA) with antibiotics (90 Units/ml penicillin·90 *μ*g/ml streptomycin, Thermo Scientific, MA, USA) and incubated at 37°C in a humidified atmosphere containing 5% CO_2_. Dulbecco's phosphate buffered saline (D-PBS) was purchased from Wako (JP).

### 2.2. Biotinylation of the Antibody

The anti-Robo1 antibody (B5209B) and control antibody (B8109B) were generated as previously described [28]. Each antibody was biotinylated according to the manufacturer's instructions for “EZ-Link Sulfo-NHS-LC-Biotin” (Thermo Scientific, MA, USA). After removing the free biotin with a PD-10 desalting column (GE Healthcare Life Sciences, UK), the number of conjugated biotins per antibody molecule was estimated using a HABA assay kit (Aproscience, JP), measuring the absorbance at 500 nm.

### 2.3. Immunotoxin Preparation and Cytotoxicity Assay of Saponin

**Figure 1:**
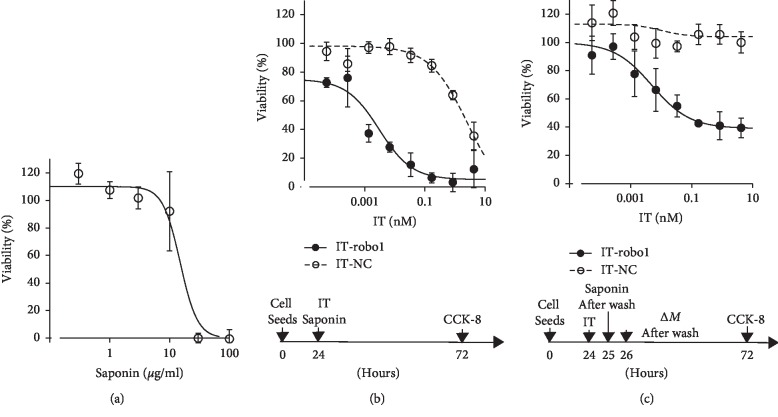
The dose-dependent cytotoxic effect of saponin on HSQ-89 cells (a). The effect of saponin on the viability of IT-Robo1 or IT-NC treated HSQ-89 cells (b, c) HSQ-89 cells were seeded at 20,000 cells/well of a 96-well plate and incubated with various concentrations (0.054 pM∼4.2 nM) of either IT-Robo1 or IT-NC together with 3.5 *μ*g/mL of saponin for 48 h. The treatment protocol for (b) continuous IT-saponin or (c) the washout experiment is designated under the respective panels. The filled circle with the solid line indicates IT-Robo1 with saponin, and the open circle with the dotted line indicates IT-NC with saponin.

A saporin-conjugated anti-Robo1 antibody (B5209B) and saporin -conjugated negative control antibody (B8109B), hereafter called IT-Robo1 and IT-NC, respectively, were prepared by incubating 121 *μ*l of 1.1 *μ*M streptavidin-saporin (Biotin-Z Internalization Kit [KIT-27-Z], Advanced Targeting Systems, CA, USA) and 138 *μ*l of 1.1 *μ*M biotinylated monoclonal antibodies for 30 min at room temperature, as described previously [14]. HSQ-89 cells were seeded at 2.0 × 10^4^ cells per well in 96-well plates and cultured overnight. On the following day, they were exposed to various concentrations (0.054 pM∼4.2 nM) of either IT-Robo1 or IT-NC (Figure 1(b)), respectively. Saponin from *Quillaja* Bark was purchased from Sigma Aldrich (MO, USA). According to the manufacturer's instruction, *Quillaja* saponin is a heterogeneous mixture of molecules varying both in their aglycone and sugar moieties, and the sapogenin content is not less than 10%. Saponin at 3.5 *μ*g/ml was added to the culture media along with IT and cultured for 48 h (Figure 1(b)). Cell viability was assessed with a CCK-8 kit (Dojindo Laboratory, JP) as described [14].

For the washout experiment (Figure 1(c)), the IT-Robo1 or IT-NC was similarly added to each well at a final concentration in a range between 0.054 pM∼4.2 nM, incubated for 1 h, washed in D-PBS and then saponin added at a final concentration of 3.5 *μ*g/ml. The cells were incubated for 1 h, washed in D-PBS, and the medium was added and incubated for 46 h. Cell viability was then similarly assessed.

### 2.4. HSQ-89 Xenograft Mice

All procedures involving mice were carried out in agreement with the protocols approved by the University of Tokyo (RAC130109-2). The semiconfluent HSQ-89 cells cultured in 10 cm Φ dishes were dissociated by trypsin, washed twice with D-PBS, and centrifuged. The cells were supplemented with D-PBS and adjusted to 2 × 10^7^ cells/ml. We mixed equal amounts of cell suspension and basement membrane matrix gel (Corning, NY, USA) [31]. We purchased 5–6 weeks old (18–20 g) male BALB/cSlc-nu/nu mice and injected them subcutaneously (SC) with 2 × 10^6^ HSQ-89 cells/200 *μ*l on their right shoulder. Water and food were provided *ad libitum*.

### 2.5. *In Vivo* IT-Robo1 with Saponin

The mice on average weighed 18–23 g (6–8 weeks old) at the start of the experiment when the tumor volume had reached 40 mm^3^. The tumor volume was calculated using the following formula: V = *π*/6 × length × width × depth [32]. IT-Robo1 at 0.1 *μ*g/D-PBS 100 *μ*l was administered intraperitoneally (IP) 5 times at 48 hr intervals. Saponin at 30 *μ*g/D-PBS 100 *μ*l was injected SC around the tumor at the same time that IT-Robo1 was administered. Acute toxicity was not exhibited in any of the groups. Mice were randomly divided into 4 groups; (1) IT-Robo1 + saponin (IT-Robo1 0.1 *μ*g/D-PBS 100 *μ*l IP + saponin 30 *μ*g/D-PBS 100 *μ*l SC), (2) IT-Robo1 only (IT-Robo1 0.1 *μ*g/D-PBS 100 *μ*l IP + D-PBS 100 *μ*l SC), (3) saponin only (D-PBS 100 *μ*l IP + saponin 30 *μ*g/D-PBS 100 *μ*l SC), and (4) PBS control (D-PBS 100 *μ*l IP + D-PBS 100 *μ*l SC). The same drug was injected four times every 48 hours, five times in total. The growth of the tumor was monitored by measuring the tumor size. When the size of the tumor reached 1,000 mm^3^ or the weight of the mice decreased drastically (i.e., a loss of more than 25% of the body weight in one week), mice were sacrificed.

### 2.6. Histological Analysis

On day 10 after the treatment was started, the representative tumors were excised from the mice after sacrifice and were put in 10% neutral buffered formalin solution (Muto Pure Chemicals, JP) for several days. After routine processing and paraffin embedding, tissues were serially sectioned. Hematoxylin-eosin (H&E) staining was used for the histological examination.

### 2.7. Data Analysis

Data are shown as means ± SD. Statistical evaluation was performed using analysis of variance (ANOVA) followed by Tukey Honest Significant Differences test. A *p* value <0.01 was taken to be statistically significant.

## 3. Results

### 3.1. Biotinylation of Monoclonal Antibodies

The anti-Robo1 monoclonal antibody (B5209B, generated in-house) [28] and control antibody B8109B were biotinylated with an EZ-Link Sulfo-NHS-LC-Biotin kit as described in Materials and Methods (M&M). The biotin molecules conjugated to each antibody were estimated by HABA assay according to the manufacturer's instructions. Both antibodies were equally biotinylated with approximately 8 biotin molecules per antibody and calculated at the absorbance value of 500 nm.

### 3.2. Cytotoxicity of Saponin

To examine the optimum concentration of saponin without cytotoxic activity, HSQ-89 cells were seeded at 2.5 × 10^3^ cells per well in 96-well plates and cultured at 37°C overnight under various concentrations of saponin. The next day, cell survival was measured by CCK-8 as described in M&M. As shown in Figure 1(a), at the concentration of 3.5 *μ*g/ml there was no significant cytotoxicity of saponin itself. On the other hand, cell death was observed over the concentration of 10.0 *μ*g/ml. Based on these results, saponin at a final concentration of 3.5 *μ*g/ml was chosen for the subsequent IT cytotoxicity experiments.

### 3.3. Cytotoxic Effects of IT-Robo1 with Saponin *In Vitro*

Saporin conjugation was carried out for both the anti-Robo1 antibody (B5209B) and the negative control antibody (B8109B) by them to a streptavidin-saporin solution, as described previously [14]. The respective conjugated antibodies are hereafter called IT-Robo1 and IT-NC. As reported previously [14], these antibodies had little effect on the viability of HSQ-89 cells, although the photochemical internalization augmented the cytotoxic effect of IT-Robo1. These results prompted us to check the effect of saponin to determine whether it facilitates the endosomal/lysosomal escape of toxin into the cytosol [18–20].

As shown in Figure 1(b), IT-Robo1 demonstrated a clearly stronger cytotoxic effect than IT-NC. However, at a concentration of 4.2 nM, IT-NC also decreased the survival rate of the cells by approximately 30% (ANOVA, *p* < 0.01) (Figure 1(b)). This means there was a nonspecific effect of IT with saponin without the formation of the antigen-antibody complex. Thus, we next performed a washout of IT and saponin from the culture medium. As shown in Figure 1(c), in the washout condition, there was no effect seen in the IT-NC control, but the cytotoxic effect remained in the IT-Robo1 treated cells. The cell viability was 40% at a concentration of IT-Robo1 4.2 nM and 100% at a concentration of IT-NC 4.2 nM (Figure 1(c)). The cytotoxic effect of IT-Robo1 was significantly greater than IT-NC (ANOVA, *p* < 0.01).

### 3.4. Antitumor Effects of IT-Robo1 with Saponin *In Vivo*

HSQ-89 cells were inoculated subcutaneously nude mice as described in M&M to check the effects of saponin on IT treatment *in vivo*. The dosage and administration route of saponin were determined as described in a previous report [25]. No acute toxicity assuming hemolysis was observed by SC injection of the *Quillaja* Bark saponin used in this study at 30 *μ*g/D-PBS 100 *μ*l.

At approximately 10 days after the inoculation, when the tumor size had reached 40 mm^3^, mice were divided into 4 groups and treated by IT in combination with saponin: (1) IT-Robo1 + saponin, (2) IT-Robo1 only, (3) saponin only, and (4) PBS control.

**Figure 2:**
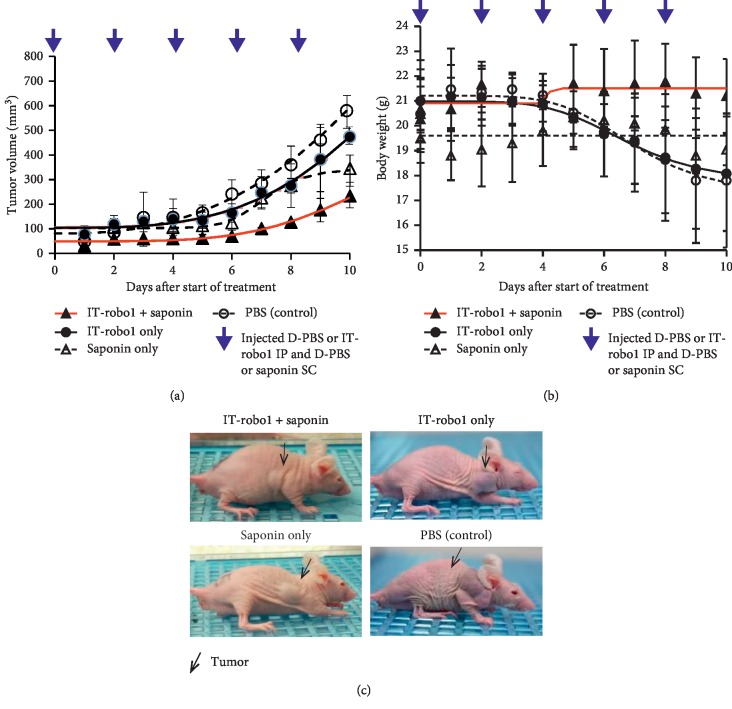
Effect of IT-Robo1 saponin treatment on xenograft on mice. HSQ-89 cells were inoculated subcutaneously on nude mice. (a) Tumor growth was treated with (1) IT-Robo1 + saponin, (2) IT-Robo1 only, (3) saponin only, and (4) PBS (control): The tumor growth in the mice treated with IT-Robo1 + saponin was inhibited compared to the other mice treated (ANOVA, *p* < 0.01). (b) The body weight reduction in the IT- Robo1 + saponin was significantly ameliorated compared to the mice receiving the other treatments (ANOVA, *p* < 0.01). (c) Images of the mice bearing the tumors treated with IT-Robo1 + saponin, IT-Robo1, saponin only, and PBS at 10 days: IT-Robo1 + saponin macroscopically delayed the progression of the tumors.

As shown in Figure 2(a), a significant reduction in tumor growth was observed in the mice in the IT-Robo1 + saponin group compared to the mice in either IT-Robo1only group or saponin only group as well as the control group (ANOVA, *p* < 0.01). A reduction in the weight of the IT- Robo1 + saponin treated group was significantly inhibited compared to the other groups (Figure 2(b)) (ANOVA, *p* < 0.01). As shown in Figure 1(c), the IT-Robo1 + saponin treated group macroscopically exhibited a delay in tumor growth.

### 3.5. Histopathological Evaluation of Tumor

**Figure 3:**
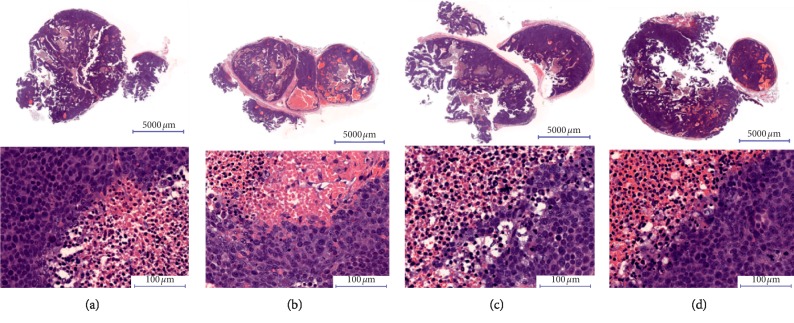
Histopathological evaluation of tumors treated with IT-Robo1 + saponin, IT-Robo1 only, saponin only, and PBS. Tumors were excised 10 days after treatment and stained with H&E; (a) IT-Robo1 + saponin, (b) IT-Robo1 only, (c) saponin only, and (d) PBS control.

In the IT-Robo1 with the saponin treatment group, there was conspicuous coagulative necrosis with granulation tissue formation inside and outside the tumor (Figure 3(a)). In the IT only treatment group, massive hemorrhagic necrosis was observed in the tumor. Broad granulation tissue formation with neovascularization was seen adjacent to the tumor (Figure 3(b)). In the saponin only treatment group, central cavity formation was seen in the tumor. The cavity was surrounded by degenerated tumor cells with pyknotic nuclei and karyorrhexis, but necrosis of tumor cells was rarely observed. Focal granulation tissue was seen adjacent to the tumor (Figure 3(c)). In the control PBS treatment group, a large cavity was observed which may have been caused by central necrosis, which suggests an extremely rapid tumor growth that exceeds the blood supply (Figure 3(d)).

## 4. Discussion

Robo1 was originally reported as a surface antigen of hepatocellular carcinoma [23]. However, ROBO1 was previously identified as a cancer suppressor gene, and growing has accumulated that Robo1 is specifically expressed in a wide range of malignant cells or neoangiogenic endothelial cells [22, 26]. It is now known as a good molecular target for cancer therapy.

Based on the possibility of endosomal release of IT, we checked the effect of saponin in this study. Saponin is known to facilitate the endosomal release of IT such that it drastically augments the cytotoxic effect of protein toxins such as saporin, a type I ribosome-inactivating protein [18, 20]. The major component of Quillaja Bark saponin is a triterpenoid saponin of which aglycone is the Quillaic acid type, and it is known to have anticancer activity [15]. We have previously confirmed the expression of Robo1 in several HNSCC cell lines [14]. Among these, HSQ-89 exhibited a level of expression similar to the hepatocellular carcinoma cell line HepG2. The anti-Robo1 monoclonal antibody B5209 that was generated in-house has been shown to have a high affinity to human Robo1 [28, 33]. The tumor uptake of ^111^In-anti-Robo1 antibody reached a maximum of 15.0 ± 0.69% ID/g at 48 h after injection and remained high for an extended period of time in HepG2 xenografts [29]. Radioactive anti-Robo1 labeled with ^90^Y has been shown to exhibit antitumor activity against small cell lung carcinoma and HepG2 xenografts [29, 30]. As previously reported, anti-Robo1 IT conjugated with saporin exerts a little cytotoxic effect on HSQ-89 cells [14]. However, photosensitizer treatment and light exposure augment the cytotoxic effect of Robo1-IT tremendously, suggesting the possibility of endosomal retention of IT [14]. The PCI technique is thus a means to achieve endosomal release of IT, but the capacity of light penetration into deep tissues is limited [34, 35].

The main activities of *Quillaja* saponins have been widely described including the antibacterial, antiviral, antifungal, antiparasitic, antitumor, hepatoprotective, and immunoadjuvant ones [17], but are too toxic to be useful in humans.

In an effort to separate the hemolysis from cancer cell killing effect, Hu et al. separated the *Quillaja* saponins fraction 21 (QS-21), a more hydrophobic fraction with an acyl chain-ASAP, and formulated it into a nanoparticle form, a killing and growth inhibiting (KGI) ASAP [16]. The ASAP's lytic effect on red blood cells was inhibited by the formulation with KGI practices, and ASAP-KGI induces cancer cell death through apoptosis [16]. Acute toxicity of *Quillaja* saponins *in vivo* was reported by Tam and Roner in 2018. They were orally administrated to newborn mice, and the LD_50_ was established at 32.5 *μ*g/mouse [36].

As shown in Figure 1(a), saponin has a dose-dependent cytotoxic effect on HSQ-89 cells, and the concentration of 3.5 *μ*g/ml was selected for further IT *in vitro* study. We have previously reported there is little effect of IT on HSQ-89 cells, and even at the maximal dose (4.2 nM) 60% of the cells were still alive [14]. In this study, the addition of saponin to the culture medium resulted in an augmentation of the cytotoxic IT-Robo1 effect on HSQ-89 cells, as its IC_50_ was several dozen pM and most cells died at around 1 nM (Figure 1(b)). At the same time, the control antibody IT-NC also displayed cytotoxic activity against HSQ-89 cells in the higher concentration range, which is considered a nonspecific saponin effect (Figure 1(b)). We utilized a washout procedure in an effort to avoid this nonspecific effect of saponin treatment. As shown in Figure 1(c), a significant cytotoxic effect of IT remained after the washout, suggesting that the antibody itself was effective. The efficacy of saponin augmentation on Ramos cells as a B-cell lymphoma treatment with saporin-Rituximab was reported by Gilabert-Oriol et al. [37].

There have been *in vivo* studies of synergistic antitumor effects with chimeric toxins, that is, saporin-EGF and saponin for epidermal growth factor receptor expressing tumor xenografts [19, 20]. We further examined the *in vivo* antitumor effect of IT-robo1 under saponin treatment using HSQ-89 cell xenograft mice. To avoid the systemic side effects of saponin such as hemolysis [16, 38], we have chosen the subcutaneous administration of saponin nearby the tumor. We have not observed any symptoms suggestive of acute adverse effects. As shown in Figure 2, the coadministration of saponin and IT-robo1 resulted in a significant reduction of tumor volume and retention of body weight. Conspicuous coagulative necrosis with granuloma formation inside or outside the tumor was observed by histopathological evaluation of the remnant tumor in the mice receiving IT-Robo1with saponin treatment, suggesting tumoricidal effects and initiation of the healing process. These results suggest a synergistic antitumor effect of IT and saponin.

IT is a potent tool in cancer therapy and there have been many trials in combination with various toxins, cancer-specific targets, and enhancers [8]. Among them, the saporin-based toxins have been shown to be of importance since saporin does not have any specific membrane receptors so, when used in combination with a targeted modality such as an antibody, they acquire specificity for cancer cells [11]. Saponins have been shown to facilitate endosomal/lysosomal escape of ribosome-inactivating proteins such as saporin used here without affecting plasma-membrane integrity [18]. The results reported here suggest that saponin facilitates the cytotoxic effect of saporin-based IT and thus widens the therapeutic window of molecular targeted treatment of cancer.

## 5. Conclusions

It is suggested that the saporin-conjugated anti-Robo1 immunotoxin comprises a novel therapeutic target in HNSCC by the application of saponin, and the drug delivery system developed here should prove to be applicable to other cancer targets.

## Data Availability

The data used to support the findings of this study are included within the article. Previously reported data were used to support this study and are available at DOI: 10.21767/2254–6081.100157. These prior studies are cited at relevant places within the text as references.
